# Perioperative complications in infant cleft repair

**DOI:** 10.1186/1746-160X-3-9

**Published:** 2007-02-05

**Authors:** Thomas Fillies, Christoph Homann, Ulrich Meyer, Alexander Reich, Ulrich Joos, Richard Werkmeister

**Affiliations:** 1 Department of Cranio-Maxillofacial Surgery, University Münster, Waldeyerstr. 30, D-48149 Münster, Germany; 2 Department of Maxillofacial and Facial Plastic Surgery, University of Düsseldorf, Moorenstrasse 5, 40225 Düsseldorf, Germany; 3 Department of Anaesthesiology, University Münster, Albert-Schweizerstr. 33, 48161 Münster, Germany; 4 Department of Oral and Maxillofacial Surgery, Central German Armed Forces Hospital, Rübenacher Str. 170, 56072 Koblenz, Germany

## Abstract

**Background:**

Cleft surgery in infants includes special risks due to the kind of the malformation. These risks can be attributed in part to the age and the weight of the patient. Whereas a lot of studies investigated the long-term facial outcome of cleft surgery depending on the age at operation, less is known about the complications arising during a cleft surgery in early infancy.

**Methods:**

We investigated the incidence and severity of perioperative complications in 174 infants undergoing primary cleft surgery. The severity and the complications were recorded during the intraoperative and the early postoperative period according to the classification by Cohen.

**Results:**

Our study revealed that minor complications occurred in 50 patients. Severe complications were observed during 13 operations. There was no fatal complication in the perioperative period. The risk of complications was found to be directly correlated to the body weight at the time of the surgery. Most of the problems appeared intraoperatively, but they were also followed by complications immediately after the extubation.

**Conclusion:**

In conclusion, cleft surgery in infancy is accompanied by frequent and sometimes severe perioperative complications that may be attributed to this special surgical field.

## Background

Surgical treatment of clefts during the infancy is not only a particular challenge for the maxillo-facial surgeon but also for the anaesthesiologist. Studies by Tiret et al. [1,2] showed that the risk of complications during the general anaesthesia is three times higher in children than in adults. From the physiological point of view, an infant differs from an older child in that most organs are still immature [3,4]. Young infants possess anatomical particularities which can cause problems during the cleft treatment. The enhanced incidence of anaesthesiological complications in children with cleft lip and palate (CLP) can be attributed to various factors such as a higher viscid airway resistance, a higher incidence of respiratory infections, nutritional deficiencies, developmental anomalies and anatomical features like micrognathia, macroglossia and jaw-bone hyoplasia. Furthermore, in cleft lip and/or palate patients the anomaly requiring surgery can be associated with one of 150 different syndromes or nonsyndromical abnormalities [3,5].

The factors influencing the overall outcome of cleft repair are multiple and complex. Timing of cleft lip and palate closure remains controversial in the literature [6]. A compromise must be made on the age at surgery and the surgical outcome concerning facial growth, scarring, speech, language development, and psychological factors [7].

Until the last decade, primary cleft operations were usually carried out in the first three years of age [6]. Today, CLP repair is done within the first 12 months of life. At this age the body weight varies between 5 and 10 kg, the whole blood volume between 400 and 700 ml. Thus, blood loss is of major concern in infant surgery.

Aspects on the time schedule for cleft surgery discussed in the literature are focused mainly on local surgical demands and outcomes, whereas only a few studies consider the occurrence of perioperative complications [8,9]. The aim of our investigation was to analyse the peri- and postoperative complications of primary cleft repair in the early stages of infancy.

## Methods

The anaesthesia protocols for 174 patients with cleft lip/palate undergoing surgery at our centre in the course of three years were reviewed. Only children younger than three years at the time of surgery were included in the study. Primary closure of the cleft lip and alveolus was usually performed at the age of three months, closure of the palate at the age of nine months. No single step surgery was performed.

The perioperative supervision included pulse oximetry, ECG, measurement of the end expiratory carbonic dioxide, blood pressure, rectal measurement of temperature and auscultation using precordial stethoscopes. Face mask ventilation was performed until the child was in deep sleep. Atropine dosed at 0.01 mg kg^-1 ^body weight and Trapanal dosed at 3–5 mg kg^-1 ^were given before orotracheal intubation was done without relaxation. Special designed tubes were used with steel strengthening inside the tube. After tube fixation and monitor complementation all children got paracetamol suppositories. The body temperature was stabilized by using warm blankets. The monitoring was continued in the recovery room. The children returned to the children's ward after their conditions were stabilized.

All surgical and anaesthesiological complications were evaluated on the basis of medical records. The complications were classified in minor or severe cases based on the classification of Cohen et al. [10] Complications were recorded as minor when the heart rate exceeded 20% or dropped below 50% at the beginning or if the loss of intraoperative body temperature was about 1°C above or 2.5°C below starting level. Decreased oxygen saturation lower than 85% and disconnection of the endotracheal tube were also considered as minor complications.

Anaesthesiological difficulties like a tube dislocation, oxygen saturation below 85% exceeding one minute, an increasing heart rate above 50% of the baseline level or lower than 80 beats per minute were recorded as severe complications. Increased body temperature by more than 2.5°C was considered as hyperpyrexia. Other severe complications were laryngospasm, bronchospasm and cardiopulmonary resuscitation. The perioperative blood loss was directly determined in 68 cases by an evaluation of the weight of compresses, instruments, sucker and suction tubes and indirectly by a measurement of the haemoglobin and hematocrit concentration one day before and 12 hours after surgery.

## Results

Cleft lip closure was performed in 73 patients, cleft palate closure in 101 patients. Additional surgery, such as myringotomy in 44% (76/174) of the patients, was performed by otorhinolaryngologists when indicated. We had minor complications in 50 out of 174 operations (28.7%). Temperature variation was found to be the most frequent complication (n = 48). Other complications such as tube disconnection (n = 1), increasing blood pressure (n = 1), reintubation (n = 1) or low oxygen saturation (n = 1) occurred rarely. Tube dislocation and hyperthermia occurred in two patients, hypothermia in one patient. Difficulties during intubation led to fiberoptic intubation in one infant, and reintubation in another. Laryngospasm and bronchospasm each occurred once. During the 174 operations 25 (14.4%) severe complications occurred in 13 patients (Table 1). Two of these 25 severe complications appeared in the group of syndromic cleft patients (2/5, Down's syndrome (two patients), De-Georgie's Syndrom, Marfan's syndrome, Pierre Robin's).

**Table 1:** Occurences of minor and severe complications (scp: syndromic cleft patient)

**minor complications**	quantity (n = 174)	**severe complications**	quantity (n = 174)
Hypothermia	15	hypothermia	1
Hyperthermia	30	Hyperthermia	2
tube disconnection	1	CPR	2
increasing blood pressure	1	tube dislocation	2
Reintubation	1	Bradycardia	5
low oxygenation	2	Iow oxygenation	5
		difficult intubation (scp)	1
		reintubation	1
		laryngospasm (scp)	1
		bronchospasm	1
50 operations	50	13 operations	25

We found a direct correlation between the occurrence of complications and the body weight at the time of operation. Complications were found in 54 % of patients weighing between 4 and 6 kg. The incidence of complications in patients with a bodyweight of more than 8 kg was found to be 26% (Table 2). Regarding the occurrence of all severe and minor complications we found no significant differences between the groups of lip closures and palate closures. Correlating with the complication rates regarding the body weight, we found 8 operations with severe complications and 5 operations with minor complications in the group of lip closures (Table 3).

**Table 2:** Coherence between body weight and anaesthesiological complication

weight (kg)	quantity (n = 174)	No complications	minor complications	severe complications
4–6	42	22/42 (52.4%)	15/42 (35.7%)	5/42 (11.9%)
6–8	52	31/52 (59.6%)	18/52 (34.6%)	3/52 (5.8%)
>8	80	58/80 (72.5%)	17/80 (21.3%)	5/80 (6.3%)

	174	111/174 (63.8%)	50/174 (28.7%)	13/174 (7.5%)

**Table 3:** Coherence between lip/palate closure and anaesthesiological complications

operation	quantity	no complications	minor complications	severe complications
lip closure	73	40/73 (54.8%)	25/73 (34.2%)	8/73 (11.0%)
palate closure	101	71/101 (70.3%)	25/101 (24.8%)	5/101 (5.0%)

Both minor and severe complications occurred mostly intra-operatively (45 minor complications, severe 9 complications). A increased number of complications were also found after the extubation. Complications in the recovery room occurred in 7 patients after the extubation.

**Figure 1:**
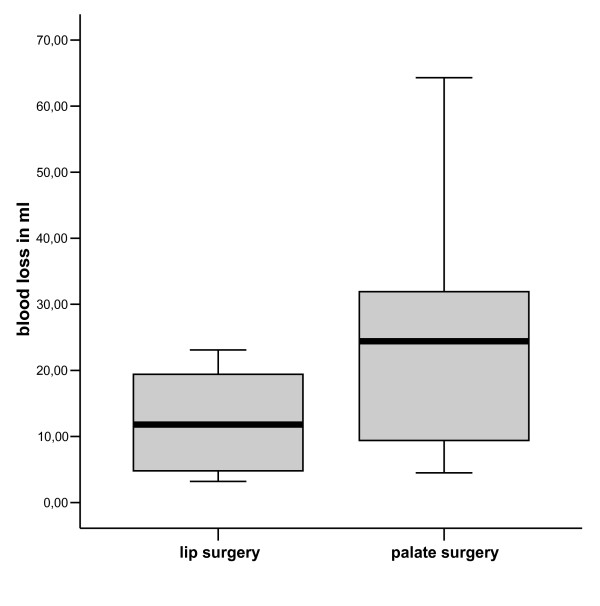
Directly measured blood loss during primary lip(n = 28) and palate repair (n = 40).

The directly measured blood loss during the primary cleft repair closure of the lips was amounted to (mean (S.D.)) 15,5 ml (12,1 ml) during closure of the lips and to 28,0 ml (19.1 ml) during closure of the palate (Figure 1). In the patient group undergoing operations of the lip (28 operations) we measured a decrease in haemoglobin concentration of 1.3 g dl^-1 ^on average in 4 patients (14.2 %) and of 1.4 g dl^-1 ^in 9 (22.5 %) patients of the group with correction of the palate (40 operations). Decreased haemoglobin concentration was found in 8 patients (21.4%) after lip closure and in 16 patients (40 %) after closure of the palate. The average decrease in haemoglobin concentration was 4.4 % below the baseline level in the patients undergoing lip repair and about 5.5% below the baseline level in patients undergoing palate repair.

## Discussion

Many concepts of cleft repair have been discussed in literature focussing mainly on the timing of cleft surgery and its long-term surgical outcome. The potential benefits of cleft closure in infants regarding developmental and social-emotional factors must be weighed against the surgical risks because the risk of early cleft repair is basically the risk of surgery in early infancy [6,10]. Our study revealed frequent complications during cleft surgery. A high number of complications were associated with the emergence and maintenance of stable upper airway during intubation, ventilation and extubation. The data of this study confirm the findings of other authors evaluating airway complications during cleft surgery [11]. Gunawardana [12] prospectively studied 800 pedriatric patients undergoing a repair of a cleft lip and palate in order to determine the factors that are predictive of difficult laryngoscopy. The occurence of a difficult laryngoscopy (Cormback and Lehane grade III and IV [13]) was found to be 3.0% in patients with a unilateral cleft lip, 45.8% in patients with a bilateral cleft lip and 34.6% in patients with retrognathia. It was demonstrated by Gunawardana [12] that in general, laryngoscopy becomes easier with increasing age (66.1% of the patients with a difficult laryngoscopy were younger than 6 months of age). As extensive clefts, retrognathia and an age of less than 6 months are associated with difficult laryngoscopy, these conditions have to be kept in mind when the anaesthetic technique is planned [12]. Van Boven thus concluded that it would be necessary to have an experienced anaesthesiologist with expertise in children's anaesthesia being supported by appropriate intra- and postoperative monitoring [4]. This is of particular importance considering the possible association of nonsyndromatic abnormalities with clefts of the lip and palate and resulting anaesthesiological complications. In 40% of the cases of the group of cleft patients with syndromic abnormalities we observed severe complications such as difficult intubation and bradycardia.

Moreover, our investigations revealed a significant number of minor and severe complications in the recovery room. For this reason we agree with Denk and Magee that specialised postoperative care with experienced medical and nursing staff is of equal importance as careful preoperative evaluation and safe intraoperative care [6].

Our investigations revealed frequent complications that may be attributed directly or partially to intra-operative blood loss. Whereas the alteration of the heart beat frequency is a direct consequence of blood loss, the lowering of the body temperature is an indirect consequence. The shortening of the duration of a cleft surgery is an important step to reduce the total loss of blood [11]. The reduction of the intra-operative blood loss is one approach to decreasing the probability and the severity of intra- and post-operative complications. A blood loss of about 50 ml during infant surgery with total patient blood volume of 400 to 700 ml can disturb the circulation, requiring a transfusion of blanked blood or plasma substitutes. A precise assessment of the blood loss is therefore vital in order to find the balance between over-transfusing and unnecessary transfusion [15,16].

An exact determination of quantity of intra-operatively lost blood is important, though methodologically difficult. Several methods to monitor perioperative blood loss have been described in literature – weighing swabs [17], colorimetry [18], osmolality dilution technique [19] and methods specifically for cleft surgery [15].

Clinical studies showed that the amount of blood loss depends on the operation technique, the surgeon's experience and the timing of cleft closure [6,16]. Scheunemann and Stellmach, for example, described an average blood loos of 32–50 ml in their patient group during an unilateral cheiloplasty. Cheiloplasty in combination with the repair of the nasal floor was associated with an average blood loss of 49–60 ml and confirmed on palatoplasty with a blood loss of about 87–129 ml [20]. Another investigation by Reinisch described an average blood loss of 30 ml during cheiloplasty [21].

In infant surgery, special consideration should be given to the fact that 50–59% of the haemoglobin is fetal haemoglobin with impaired oxygen emission despite generally high haemoglobin concentrations in the infant period. A newborn infant is therefore dependent on higher haematocrit [22]. It has to be kept in mind that in the first 3 months of life the normal haemoglobin concentration decreases to low values (trimenon anaemia) because fetal haemoglobin decreases and is only slowly replaced by adult haemoglobin [16].

In this study, the blood loss was first directly quantified. Additionally the haemoglobin and haematocrit concentration were measured before and 12 hours after surgery. In our patient group blood loss was higher after the repair of the palate than after closure of the lip. No blood transfusion was necessary. We found an increased incidence of complications in dependence on the body weight at the time of operation. In accordance to our findings Wilhelmsen and Musgrave found that a body weight of more than 5 kg, haemoglobin of more than 10 g dl^-1 ^and additionally a white blood count of less than 10000 μl^-1 ^was associated with less risk of complications to the factor of 5 [23].

Our study revealed that the risk of perioperative complications was found to be correlated to the body weight at the time of the surgery. Substantially, the perioperative complication concern anaesthesiological complications in cleft repair. No severe surgical complication as fulminant blood loss was found.

## Conclusion

In view of today's multitude of time-related concepts of cleft surgery investigations are required searching for the optimal moment for a cleft repair – with low severe perioperative complication rates but favourable functional and aesthetic results.

## Competing interests

The author(s) declare that they have no competing interests.

## Authors' contributions

TF: Project planning, data analysis and writing of the manuscript

CH: Project planning, data analysis and writing of the manuscript

UM: Writing of the manuscript, critical appraisal of the manuscript

AR: Project planning, critical appraisal of the manuscript

UJ: Critical appraisal of the manuscript

RW: Project planning, critical appraisal of the manuscript
